# Head to Toe: A Perioperative Surgical Home Approach to Sepsis and Airway Obstruction in a Geriatric Patient

**DOI:** 10.7759/cureus.76040

**Published:** 2024-12-19

**Authors:** Holim Lee, Justin Calvert, Mira Bishawi, George Wong, Leonard J Soloniuk, Gary Stier

**Affiliations:** 1 Internal Medicine, Chungnam National University College of Medicine, Daejeon, KOR; 2 Department of Anesthesiology and Perioperative Medicine, Riverside University Health System Medical Center, Moreno Valley, USA; 3 Department of Anesthesiology, Loma Linda University Medical Center, Loma Linda, USA; 4 Department of Gynecology and Obstetrics, Loma Linda University Medical Center, Loma Linda, USA

**Keywords:** case report, interdisciplinary care, perioperative surgical home, risk stratification, urgent surgery

## Abstract

The perioperative surgical home (PSH) is a care delivery model designed to improve the perioperative and long-term outcomes of patients undergoing surgery by promoting holistic care and seamless cooperation between different services and subspecialties. An aging population and increased surgical complexity have led to renewed interest in PSH models. An 86-year-old female with diabetes and critical limb ischemia presented with sepsis due to right calcaneal gangrene. A structured, comprehensive evaluation by the inpatient PSH service identified perioperative concerns including malnutrition and elevated cardiac risk and led to the discovery of an airway-compromising mass. A coordinated care plan, devised by the PSH team resulted in a joint operation by otolaryngology and orthopedic surgery departments, thus avoiding what would have been an otherwise unexpected difficult airway, and likely complications. Our case highlights the explicit and implicit benefits of interdisciplinary care coordinated by an inpatient PSH service, which includes risk stratification, comprehensive evaluation, and prevention of complications.

## Introduction

PSH is a care delivery model developed by the American Society of Anesthesiologists around 2011 to improve the perioperative and long-term outcomes of patients undergoing surgery by promoting, among others, seamless cooperation between different services and subspecialties [1].

PSH is extremely relevant in today’s healthcare landscape, especially concerning geriatric populations. People over the age of 80 are the fastest-growing population and are expected to comprise 8% of the U.S. population by the year 2050 [2]. Perioperative care of geriatric patients is complex, with nearly 50% of those aged 80 and above, having three or more comorbidities. Successful perioperative management of these medically complex patients is dependent on care coordination by interprofessional teams.

The PSH model has proven successful in large urban health centers, where it has resulted in fewer surgery cancellations, reduced operating room time, shorter length of stay (LOS,) and reduced readmission rates [3]. Other organizations, including Veterans Affairs hospitals, have also reported positive changes after adopting the PSH approach [4,5].

This case report describes how the implementation and utilization of a structured PSH care model focusing on inpatient preoperative risk stratification, management, and care coordination improved the care of a medically complex female patient in her 80s presenting for an urgent source-control surgery with a discovery of airway compromise.

This study was conducted at Riverside University Health System Medical Center, with all authors either affiliated with the institution or completing a rotation there as an international medical student. All other affiliations were listed for full disclosure. All authors contributed equally to the work.

## Case presentation

**Figure 1:**
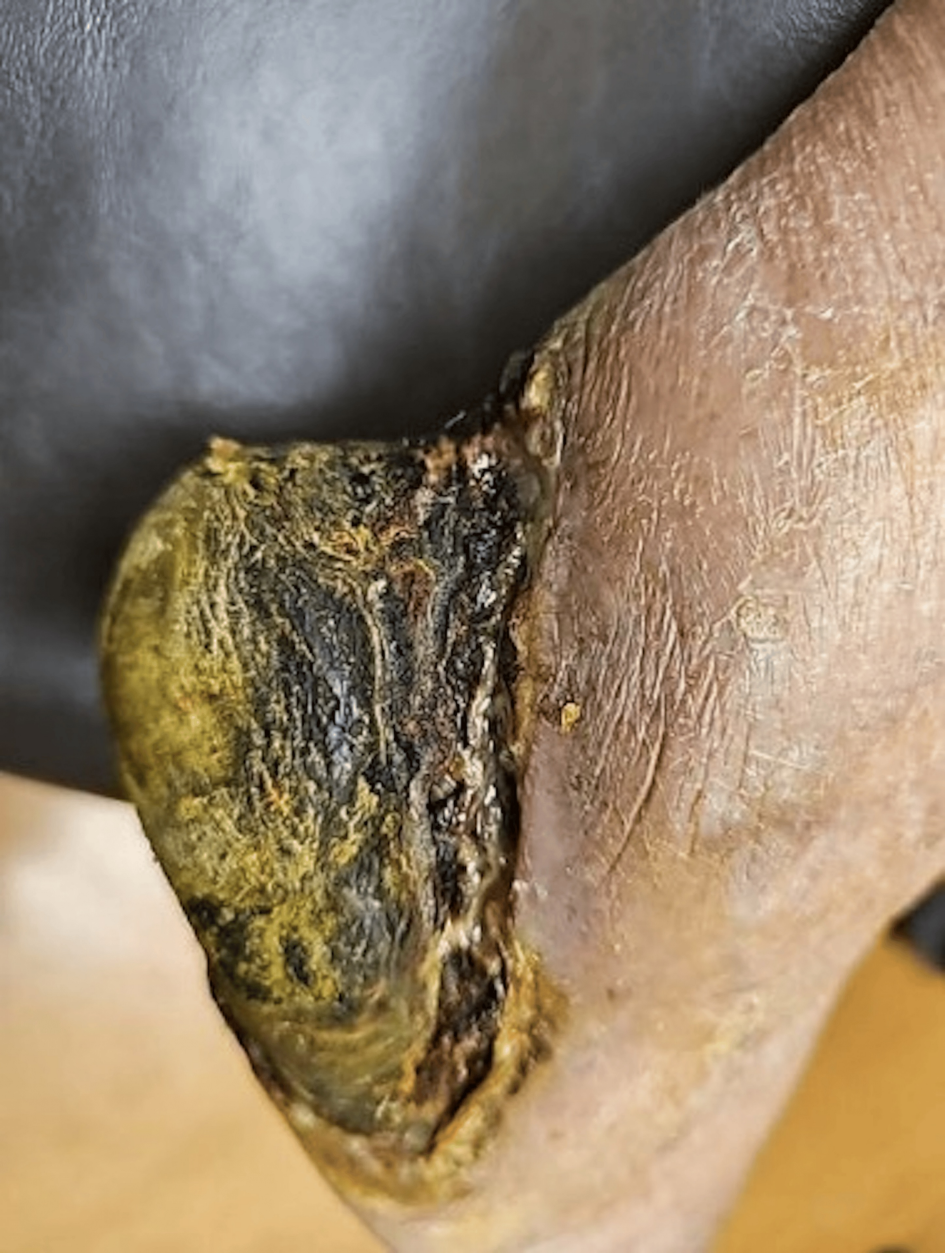
Photo of the patient's right heal demonstrating dry gangrene.

An 86-year-old female with a past medical history of insulin-dependent diabetes, peripheral arterial disease with previous left below-knee amputation (BKA,) chronic kidney disease stage 3, hypertension, and dementia presented to Riverside University Health System (RUHS) Emergency Department following four days of progressive right foot pain and purulent discharge. The patient was brought from Mexico by her family less than a year ago due to suboptimal diabetes management. At presentation, she was found to be septic, and an evaluation of her foot wound revealed dry gangrene (Figure 1) and tissue loss consistent with critical limb ischemia. She was admitted for the management of sepsis and an urgent surgical amputation for source control.

Patient assessment and risk stratification

Due to the patient’s age and other comorbidities, a consultation request was placed for preoperative assessment by the RUHS inpatient PSH service. The PSH team performed a structured comprehensive assessment including the incorporation of the following risk assessment tools:

*Duke Activity Status Index *(*DASI*)* and the Revised Cardiac Risk Index *(*RCRI*)

The Duke Activity Status Index (DASI) [6,7], a validated tool to estimate activity level, was utilized to obtain the patient's DASI score; her metabolic equivalent of the task was estimated to be 2.74, which corresponds to a New York Heart Association functional class of III [9]. Additionally, as per the Revised Cardiac Risk Index (RCRI), her RCRI Class risk score was II, and it is known that due to the changed healthcare landscape, RCRI can underestimate cardiac risk [10]. Given her intermediate cardiac complication risk, a proBNP level was obtained and found to be elevated at >12,000 pg/mL. A transthoracic echocardiography revealed a preserved left ventricular ejection fraction of 65-70% with moderate mitral stenosis and mild mitral regurgitation. Additionally, the patient’s American Society of Anesthesiologists Physical Status was Class IV due to sepsis in addition to her chronic conditions.

National Surgical Quality Improvement Program (NSQIP) Surgical Risk Calculator

**Figure 2:**
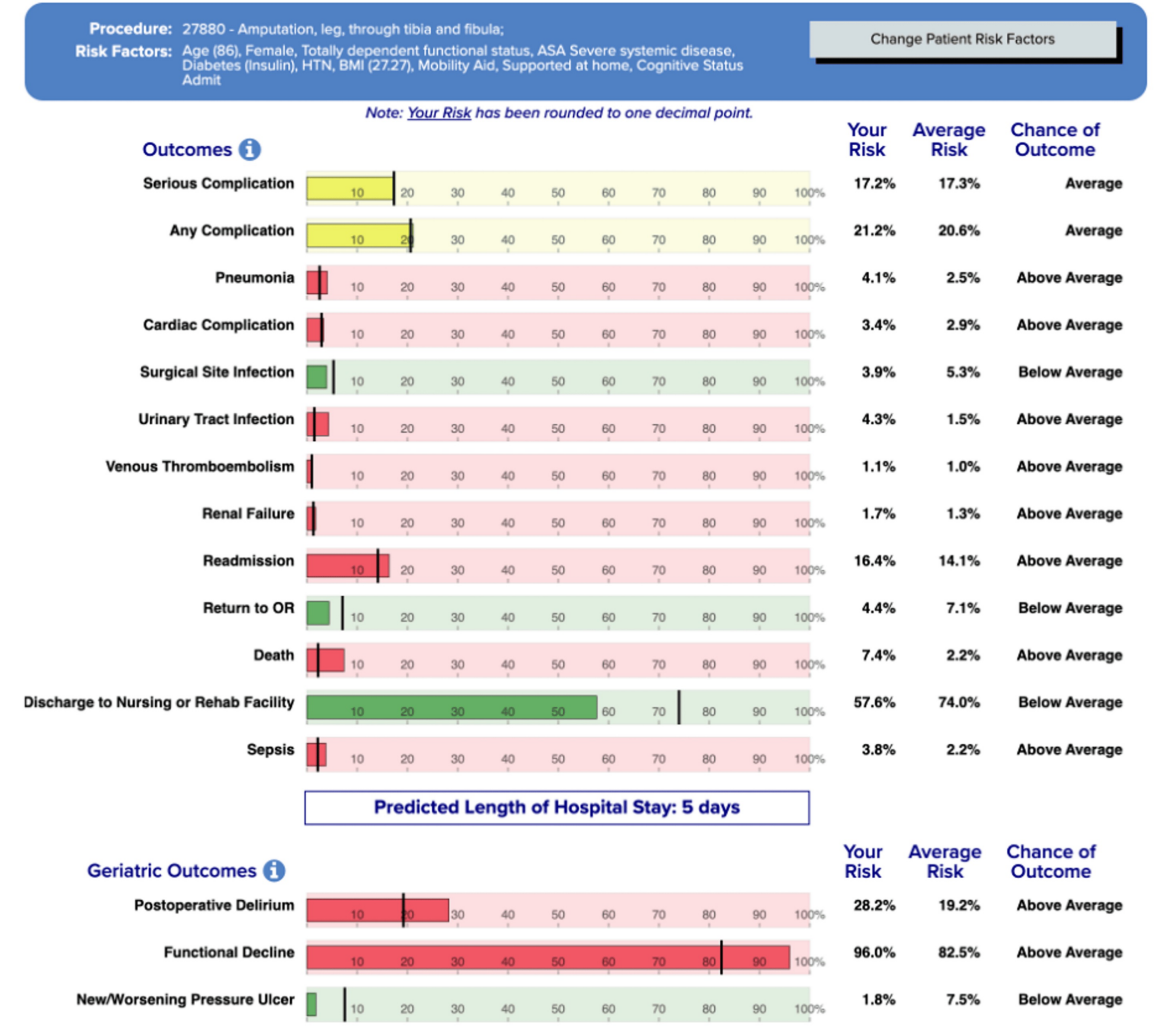
The NSQIP risk calculation for the patient, including risk of cardiac complication NSQIP: The National Surgical Quality Improvement Program [11].

The patient’s NSQIP risk [11] was significant for postoperative pneumonia, hospital readmission, death, and sepsis; her cardiac risk was estimated at 3.4%. In addition, the geriatric risks were high for postoperative delirium and functional decline (Figure 2).

Global Leadership Initiative on Malnutrition Assessment

Despite her body mass index of over 25.5 kg/m^2^, malnutrition was diagnosed as per the Global Leadership Initiative on Malnutrition Assessment [12] due to recent weight loss, chronic inflammation, and hypoalbuminemia.

Comprehensive History and Chart Review

**Figure 3:**
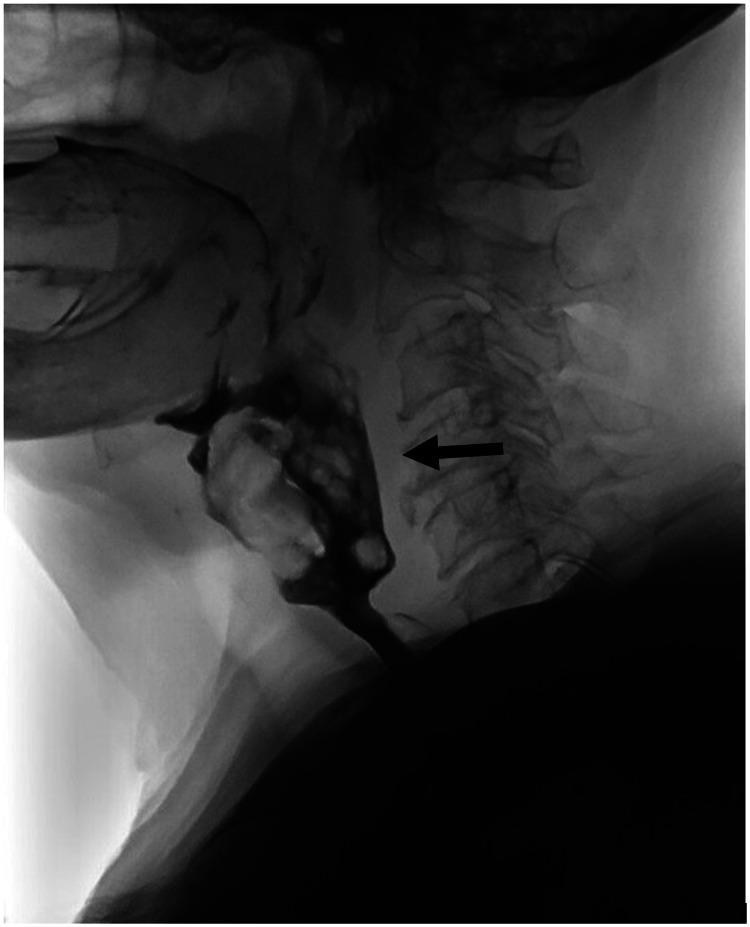
A fluoroscopic swallowing study demonstrating an obstructing hypopharyngeal mass (indicated by arrow).

**Figure 4:**
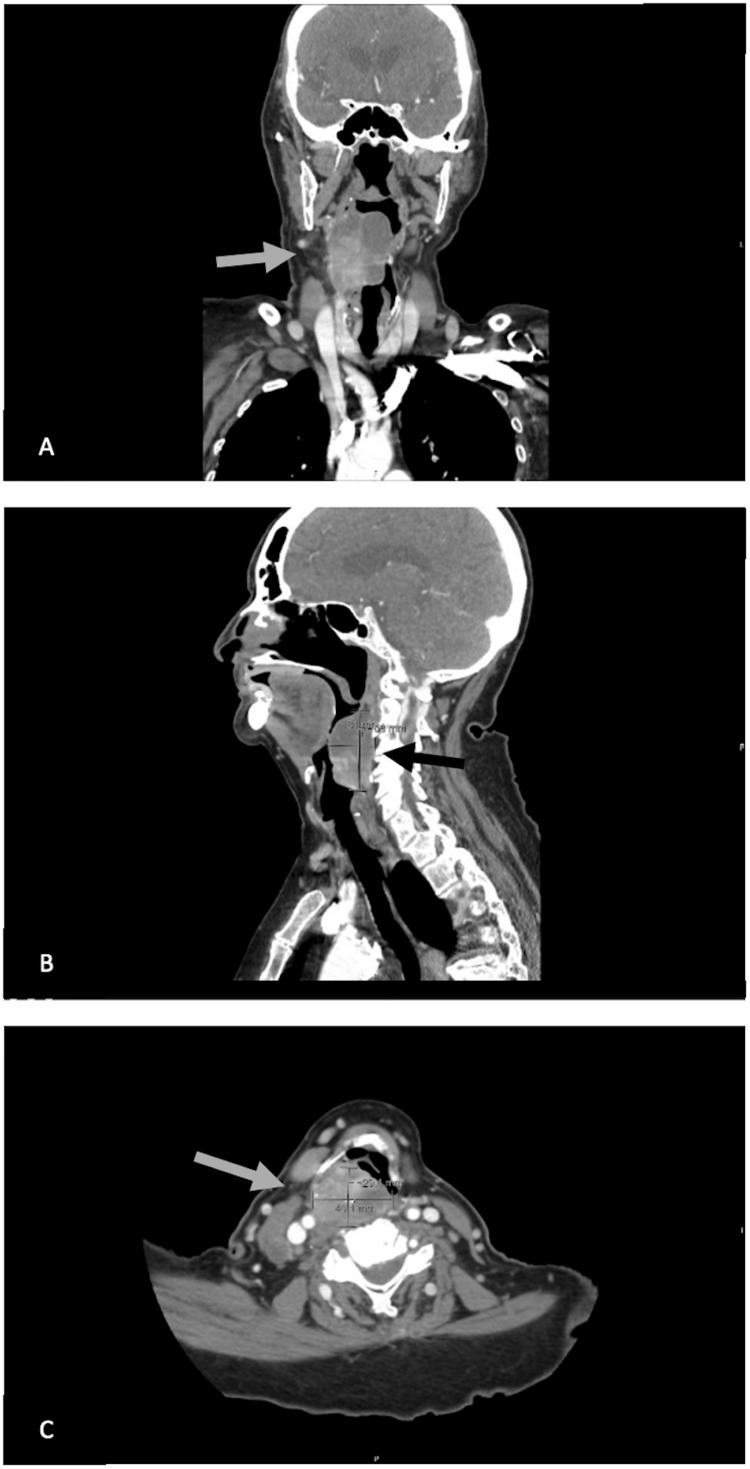
A CT scan demonstrating a 5x5x3 cm mass (indicated by arrows) superior to the larynx with lymphadenopathy in the (A) coronal, (B) sagittal, and (C) axial views. CT: Computed tomography

Recent outpatient geriatrics clinic notes described symptoms of dysphagia and concern for aspiration. An inpatient videofluoroscopic swallowing study was obtained and revealed an obstructing hypopharyngeal mass (Figure 3). A subsequent computed tomography (CT) scan ordered by the PSH team confirmed the 5x5x3 cm mass superior to the larynx and lymphadenopathy (Figure 4).

Operative course and postoperative management

Based on the information from the preoperative assessment, otolaryngology was consulted, and a combined operation with the right BKA was agreed upon. This operation was immediately preceded by an awake tracheostomy and tumor biopsy performed by the otolaryngology department.

The two-phase surgery was performed on hospital Day 3. The intraoperative course was unremarkable, with a total operative time for the combined procedures of just under three hours. Phenylephrine infusion titrated to a maximum rate of 100mcg/min was used intraoperatively and was weaned off prior to emergence.

Postoperatively, the patient was transferred to the intensive care unit (ICU) for the management of her new tracheostomy. The patient was hemodynamically stable on transfer; However, low-dose vasopressors were later required. Her ICU course was complicated by acute kidney injury, elevated leukocyte count, and persistent respiratory distress. On postoperative Day 7, she was weaned to a tracheostomy mask, and an initial tracheostomy tube change was performed. Then, another respiratory decline occurred with a renewed need for mechanical ventilation. Biopsy results returned and showed metastatic papillary thyroid cancer. Her lower extremities showed poor wound healing, and subsequent arterial angiography revealed severe stenosis of multiple right lower extremity arteries. Therefore, on postoperative Day 28 from her initial operation, the patient underwent bilateral iliac artery endovascular revascularization with endarterectomy and stenting.

Ten days after the second operation, the patient was discharged to a long-term acute care hospital with an ultimate plan for outpatient revisionary amputation pending improved nutritional status.

## Discussion

Implementation of PSH at RUHS Medical Center

According to the PSH framework, a physician-led, interdisciplinary team manages a patient's perioperative care experience. These coordinated efforts of the medical and surgical care teams have led to several advantages for patients, such as shorter LOS, reduced complications, decreased readmissions, and improved post-discharge quality of life [13].

Importantly, the implementations of PSH often occur piecemeal with initiatives focused on institutional needs [14]. At RUHS, our PSH program began with the establishment of an inpatient consultation service for medically complex patients. The objectives were to risk-stratify, determine if any medical comorbidities required further workup, and determine whether the patient could benefit from preoperative treatment or optimization. Later expansions include evidence-based intraoperative management strategies, the launch of an outpatient optimization clinic, and monthly interdisciplinary rounds with various surgical services.

Benefits and limitations of the PSH involvement

Regarding the patient’s chronic conditions, recommendations were given to optimize her blood pressure and blood glucose; nutritional recommendations, including further testing, consultations, and dietary supplementation, were ordered; and medications were recommended to address her pain, delirium risk, and anticoagulation needs.

The discovery of this patient’s hypopharyngeal mass during the preoperative consultation prevented an unexpected discovery in the OR. Without the PSH consult, either a same-day cancellation or worse, an unexpected airway compromise would have likely occurred. Instead, by planning a preceding tracheostomy and performing a biopsy of the mass at the same time, the possibility of airway compromise was mitigated, and the need for a separate otolaryngologic procedure was avoided.

The major limitation of our case is that a patient-centered preoperative workup was performed, and the problems were identified in advance, but the patient’s postoperative course was extensive. Despite our efforts, there were additional ICU transfers for respiratory distress and two rapid response calls outside the ICU. In addition to her chronic conditions, the patient’s American Society of Anesthesiologists Physical Status was Class IV due to sepsis. In fact, the patient’s NSQIP Surgical Risk Calculator predicted an above-average risk of postoperative adverse outcomes, which included death.

However, prior research has shown that the rate of rapid response calls decreased for surgical patients after the implementation of PSH models [5]. Considering the patient’s right BKA for source control was inevitable, a complicated postoperative course should not discourage the implementation of PSH.

An appointment to our outpatient perioperative optimization clinic would have greatly benefited this patient before the limb deteriorated into a critical state. There is evidence that preoperative nutritional optimization strongly influences postoperative outcomes, with malnourished surgical patients having significantly higher postoperative mortality, morbidity, length of stay, readmission rates, and increased hospital costs [15]. The patient’s frailty and malnutrition could have been managed earlier, increasing the possibility of a faster recovery and a better disposition.

## Conclusions

This case report highlights the utility of an inpatient PSH consultation in a medically complex geriatric patient with an urgent need for surgery. It seeks to motivate other institutions to adopt a similar implementation strategy. Furthermore, it outlines various tools and strategies that can be used in their PSH initiatives.
